# Supplementary material for: A system-wide snapshot: A multi-campus survey of open source contributors at the University of California
Source: PLoS One. 2026 Jun 5;21(6):e0348894. doi: 10.1371/journal.pone.0348894 (PMC13241014; doi:10.1371/journal.pone.0348894)
Supplement: S1 File — Survey instrument in PDF format. See Materials and methods for a description of survey flow. (PDF) [file pone.0348894.s001.pdf]

UC OSPO Network Survey

Start of Block: Consent

Q0. This survey is being conducted by the UC Open Source Program Office Network, a group that is working to establish a network of collaborative Open Source Program Offices (OSPOs) at all UC campuses. Your participation in this study is voluntary.

This study has two main objectives: first, to assess how an OSPO on your campus can support or enhance your open source community; and second, to examine the landscape of open source repositories developed by UC researchers, with the additional goal of creating a resource to showcase UC's open source projects.

This online survey will take approximately 10-15 minutes to complete. The survey is anonymous; however, it will provide the opportunity to share your email and the username(s) associated with your open source repositories. This information is not required to complete the survey. If you choose to provide this information, it will not be associated with your other survey responses. When the survey results are published, they will be published in aggregate with all identifying information removed.

Questions about this initiative may be directed to ospo@library.ucsb.edu. Thank you for your time.

Your input is invaluable as we strive to better understand and support the open source ecosystem within our academic community.

☐ I understand and agree.

End of Block: Consent

Start of Block: Affiliation

Q1. With which UC campus are you primarily affiliated?*
This question is required.

- ◯ UC Berkeley
- ◯ UC Davis
- ◯ UC Irvine
- ◯ UC Los Angeles
- ◯ UC Merced
- ◯ UC Riverside
- ◯ UC San Diego
- ◯ UC San Francisco
- ◯ UC Santa Barbara
- ◯ UC Santa Cruz
- ◯ I'm not affiliated with UC
- ◯ Other UC

Display this question:

If With which UC campus are you primarily affiliated? = I'm not affiliated with UC

This survey is for affiliates of the University of California. For open source support, please reach out to your campus' library, IT department, and/or technology transfer office. Thank you for your interest in the UC OSPO network. Please proceed to finish the survey.

Skip To: End of Survey If This survey is for affiliates of the University of California. For open source support, please re... Displayed

End of Block: Affiliation

Start of Block: Introduction

Please respond to this survey in your capacity as a professional and/or student at UC.

Definitions:

- **"Open source"** refers to software or hardware products whose source code and/or designs are publicly accessible, allowing anyone to study, modify, and distribute them. Popular examples include the Linux kernel, the Python language, the R language, Jupyter, Firefox, Zotero, WordPress, Arduino, and many more.
- **"Contributions"** can be technical or non-technical, including but not limited to code, bug reports, advice, documentation, participation in discussions, graphic design, translations, donations, or administrative support.

Q2. How important would you say open-source projects are to your...?*

	Not at all important	Slightly important	Moderately important	Important	Very important	Non-applicable
Research	○	○	○	○	○	○
Teaching	○	○	○	○	○	○
Learning	○	○	○	○	○	○
Professional Development	○	○	○	○	○	○
Job (for non-research staff)	○	○	○	○	○	○

Q3. Please indicate your status as an open source contributor.*
Note: "Contributions" can be technical or non-technical, including but not limited to code, bug reports, advice, documentation, participation in discussions, graphic design, translations, donations, or administrative support.

	True	False
I have contributed to open-source projects in the past.	○	○
I would like to contribute to open-source projects in the future.	○	○

Warning: Please review your selections. Once you proceed, you will not be able to return to this page.

End of Block: Introduction

Start of Block: For contributors

Q4. Which of these open source contributor roles has ever applied to you?*
Select all that apply:

- ☐ **Maintainer**: Authoring much or most of the codebase/designs for at least one open-source software/hardware product intended for public reuse.
- ☐ **Contributor**: Contributing relatively small amounts of code or hardware design, for example by fixing bugs or adding new features.
- ☐ **Bug/Issue Reporter**: Reporting bugs or issues, or requesting new features.
- ☐ **Supervisor**: Guiding the strategic vision of at least one open-source project, and/or acquiring funding for it.
- ☐ **IT/Systems administrator**: Providing technical infrastructure for open source developers.
- ☐ **UI/UX Designer**: Creating user-centered interfaces and experiences for digital products.
- ☐ **Community Manager**: Engaging with the open source community, facilitating communication between developers, stakeholders, and users, and encouraging new contributions.
- ☐ **Technical support**: Assisting users with software/hardware issues by providing technical help through forums, emails, or other support channels.
- ☐ **Educator**: Creating documentation, tutorials, articles, or other materials that help people use one or more open-source tools.
- ☐ Other (Please specify. Multiple answers should be comma-separated.)
  ____

Q5. How frequently have you contributed to projects of the following size?*
For each project size, please answer relative to the other sizes. For instance, the category you contribute to most should be "Relatively frequently", even if you don't make open source contributions very often.

	Never	Relatively infrequently	Occasionally	Relatively frequently
Small: tens of users	○	○	○	○
Medium: hundreds of users	○	○	○	○
Large: Thousands of users or more	○	○	○	○

Q6. Why do you contribute to open-source projects?*
Please select all that apply:

- ☐ Developing open-source products is part of my job
- ☐ To improve the tools in my field
- ☐ To customize existing tools for my specific needs
- ☐ To build a network of peers
- ☐ To give back to the open source community
- ☐ To improve my skills
- ☐ Because it's fun
- ☐ Other (Please specify. Multiple answers should be comma-separated.)

____

Q7. What category(ies) of open-source projects have you contributed to?*
Please select all that apply:

- ☐ **Hardware**: Physical devices used for research (such as cameras, robots, or behavioral rigs), and associated code.
- ☐ **Applications**: Standalone software systems designed to be used as-is.
- ☐ **Plug-ins or extensions**: Add-ons, extensions, or modules that extend the functionality of existing software or platforms.
- ☐ **Libraries, packages, or frameworks**: Reusable code that enhances the functionality of programming languages or other packages and that is distributed through established community mechanisms.
- ☐ **Automation scripts**: Scripts or tools to automate workflows, processes, or repetitive tasks within a specific environment or project.
- ☐ **Website code**: Either back-end (e.g., database integration, server-side functionality) or front-end website components (e.g., layout, navigation, interactive features).
- ☐ Other (Please specify. Multiple answers should be comma-separated.) ____

Q8. Where have you shared the code and/or hardware designs for your open-source projects?*
Please select all that apply:

- ☐ Bitbucket
- ☐ Codeberg
- ☐ Dataverse
- ☐ Dryad
- ☐ Figshare
- ☐ Gitea
- ☐ GitHub
- ☐ GitLab
- ☐ Launchpad
- ☐ Mendeley Data
- ☐ OSF (Open Science Framework)
- ☐ SourceForge
- ☐ Thingiverse
- ☐ Vivli
- ☐ Zenodo
- ☐ A custom website (e.g. a lab website)

☐ In the supplementary components of a journal article

☐ Other (Please specify. Multiple answers should be comma-separated.)

____

Q9. How frequently have you encountered the following challenges while working on open-source projects?*

	Never	Rarely	Occasionally	Frequently	Always	Non-applicable
Limited time for writing new code	○	○	○	○	○	○
Limited time for writing documentation	○	○	○	○	○	○
Managing issues and pull requests	○	○	○	○	○	○
Attracting users and/or contributors	○	○	○	○	○	○
Receiving recognition for my contributions	○	○	○	○	○	○
Finding and hiring qualified personnel	○	○	○	○	○	○
Managing security risks	○	○	○	○	○	○
Finding a community of peers who share my interests	○	○	○	○	○	○
Finding mentors	○	○	○	○	○	○
Finding time to educate myself	○	○	○	○	○	○
Identifying helpful educational resources	○	○	○	○	○	○
Navigating licensing and other legal	○	○	○	○	○	○

issues						
Identifying potential funding sources for my open source projects	○	○	○	○	○	○
Securing funding for my open source projects	○	○	○	○	○	○

Q10. How useful would the following kinds of campus-level assistance be to you?*

	Not very useful	Useful	Very useful	Non-applicable
Access to free, feature-rich computing environments	○	○	○	○
Assistance promoting your open-source projects	○	○	○	○
Assistance creating (i.e. Docker) containers for your open-source software	○	○	○	○
Assistance writing documentation	○	○	○	○
An open source discussion group and learning community	○	○	○	○
Assistance with event planning, i.e. conferences and hackathons	○	○	○	○
A mentor/mentee program	○	○	○	○
Educational materials and workshops on programming languages, popular packages, etc.	○	○	○	○
Legal and licensing support	○	○	○	○
Assistance building industry partnerships	○	○	○	○

Dedicated grants for open-source project sustainability	○	○	○	○
Assistance identifying potential funding sources	○	○	○	○

Q11. If you had to choose one item from the list above as potentially the most useful to you, which would you choose?*

- ◯ Access to free, feature-rich computing environments
- ◯ Assistance promoting your open-source projects
- ◯ Assistance creating (i.e. Docker) containers for your open-source software
- ◯ Assistance writing documentation
- ◯ An open source discussion group and learning community
- ◯ Assistance with event planning, i.e. conferences and hackathons
- ◯ A mentor/mentee program
- ◯ Educational materials and workshops on programming languages, popular packages, etc.
- ◯ Legal and licensing support
- ◯ Assistance building industry partnerships
- ◯ Dedicated grants for open-source project sustainability
- ◯ Assistance identifying potential funding sources

Q12. Are there any other challenges you've encountered in open source, or types of support that you would find helpful?

Q13. The UC OSPO Network is building a database of publicly available open source repositories developed at UC, so that we may better understand the nature and extent of UC's open source contributions. To help with this effort, please add your username(s) on all version-controlled code-hosting platforms that you use, such as GitHub or GitLab.
Do not include data repositories such as Zenodo, Dryad, Figshare, etc.
Please format response as <platform: username>, and separate multiple values with a comma. For example, "GitHub: jane_smith42, GitLab: janeqsmith"
The information provided will not be associated with individual responses.

____

Q14. The UC OSPO network is planning to build a public online catalog to showcase open-source repositories developed at UC. If you are interested in talking with us about potentially having one or more of your repositories featured in this resource, please check the box below.

▢ Yes, please contact me about potentially featuring my work in a UC software catalog.

Display this question:

If The UC OSPO network is planning to build a public online catalog to showcase open-source reposito... = Yes, please contact me about potentially featuring my work in a UC software catalog.

Please enter your email here.*

____

Warning: Please review your selections. Once you proceed, you will not be able to return to this page.

End of Block: For contributors

Start of Block: For future contributors

Q15. What would make you more likely to participate in OSS projects?*
Please select all that apply.

- ☐ Access to free, feature-rich computing environments
- ☐ An open source discussion group and learning community
- ☐ Accessible conferences or hackathons
- ☐ A mentor/mentee program
- ☐ Educational materials and workshops on programming languages, popular packages, etc.
- ☐ Legal and licensing support
- ☐ Job/internship opportunities at other academic institutions
- ☐ Networking opportunities with industry
- ☐ Dedicated grants for open-source project sustainability
- ☐ Assistance identifying potential funding sources
- ☐ Other (Please specify. Multiple answers should be comma-separated.) ____

End of Block: For future contributors

Start of Block: Demographics and contact information

Q16. Are you...*

- ◯ Undergraduate
- ◯ Grad Student
- ◯ Post-Doc
- ◯ Faculty
- ◯ Other research staff (e.g., research scientist, research software engineer)
- ◯ Non-research Staff

Display this question:

If Are you... = Undergraduate*

Or Are you... = Grad Student*

Or Are you... = Post-Doc*

Or Are you... = Faculty*

Or Are you... = Other research staff (e.g., research scientist, research software engineer)*

Q17. With what domain do you most identify?*

▼

Humanities

Life Sciences

Humanities and Computational Sciences

Physical Sciences

Social sciences

Display this question:

If Are you... = Undergraduate*

Or Are you... = Grad Student*

Or Are you... = Post-Doc*

Or Are you... = Faculty*

Or Are you... = Other research staff (e.g., research scientist, research software engineer)*

Q18. What is your primary field? (e.g. astrophysics, neuroscience)
One response is preferred, but if multiple are needed, please separate with commas.

____

Display this question:

If Are you... = Non-research Staff*

Q19. What is your primary department or work area?*

◯ Academic and Research Support (includes research administration, libraries, and instructional design)

◯ Administration and General Operations

◯ Admissions and Enrollment Services

◯ Alumni Relations and Development

◯ DevOps or System Administration

◯ Diversity, Equity, and Inclusion (DEI)

◯ Facilities and Maintenance

◯ Finance (includes accounting, budgeting, and procurement)

◯ Health and Wellness Services

◯ Human Resources

◯ Information Technology (IT)

◯ Marketing and Communications

◯ Student Affairs and Services

◯ Other (Please specify. Multiple answers should be comma-separated.)
____

Q20. Let's keep in touch!
Your email will not be associated with your individual responses.

- [ ] I'd like to be receive periodic announcements from my campus' Open Source Program Office.

- [ ] I'd like an invitation to the UC Tech Slack Workspace, so that I can join the #open-source channel.

Display this question:

If Let's keep in touch! Your email will not be associated with your individual responses. = I'd like to be receive periodic announcements from my campus' Open Source Program Office.

Or Let's keep in touch! Your email will not be associated with your individual responses. = I'd like an invitation to the UC Tech Slack Workspace, so that I can join the #open-source channel.

Please enter your email to keep in touch.*

____

End of Block: Demographics and contact information
