# Supplementary material for: A system-wide snapshot: A multi-campus survey of open source contributors at the University of California
Source: PLoS One. 2026 Jun 5;21(6):e0348894. doi: 10.1371/journal.pone.0348894 (PMC13241014; doi:10.1371/journal.pone.0348894)
Supplement: S1 Fig — (A) Total number of aspiring and experienced contributors, for each job category. (B) Percent of aspiring and experienced contributors, for each job category. (PDF) [file pone.0348894.s002.pdf]

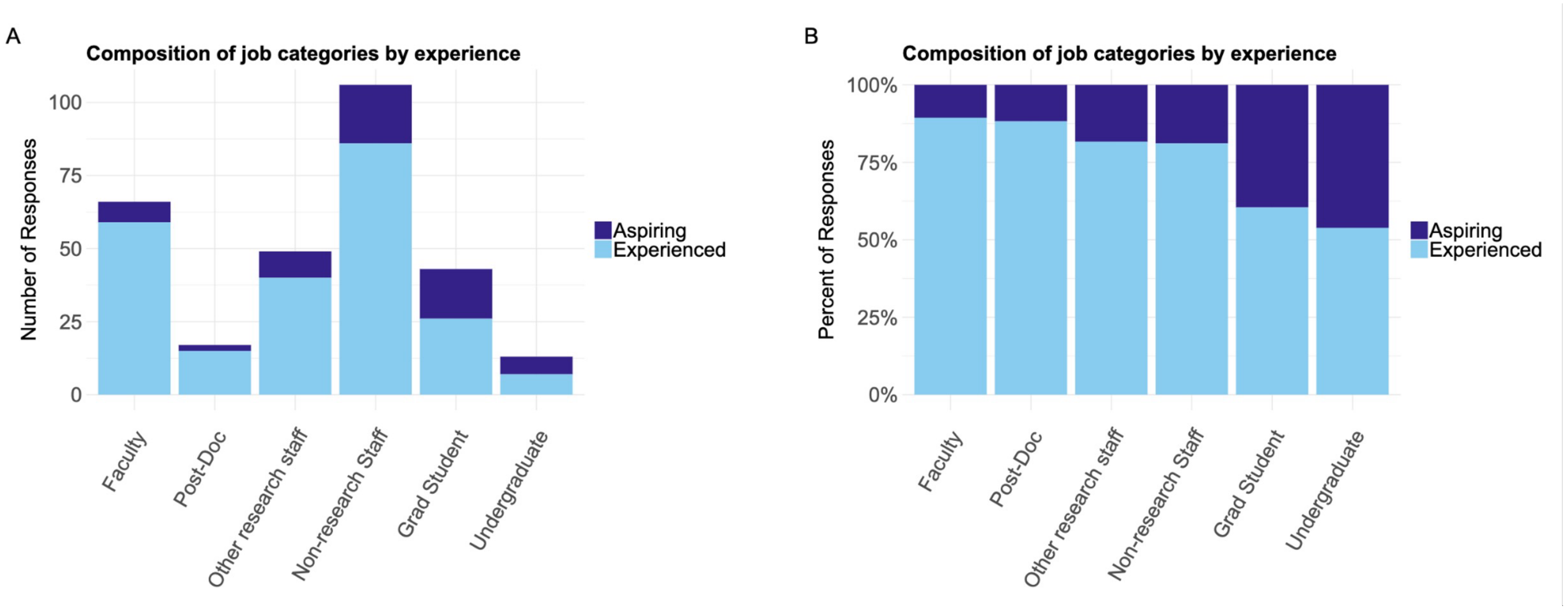

S1 Fig. Composition of job categories by open source contribution experience. (A) Total number of aspiring and experienced contributors, for each job category. (B) Percent of aspiring and experienced contributors, for each job category.
