# Supplementary material for: A system-wide snapshot: A multi-campus survey of open source contributors at the University of California
Source: PLoS One. 2026 Jun 5;21(6):e0348894. doi: 10.1371/journal.pone.0348894 (PMC13241014; doi:10.1371/journal.pone.0348894)
Supplement: S2 Fig — Scatterplot showing, for each UC campus, the approximate size of the campus community (staff and students, based on data from the UC Information Center), versus the number of responses received from that campus in this survey. The coefficient of determination for this relationship is shown in the top-right. (PDF) [file pone.0348894.s003.pdf]

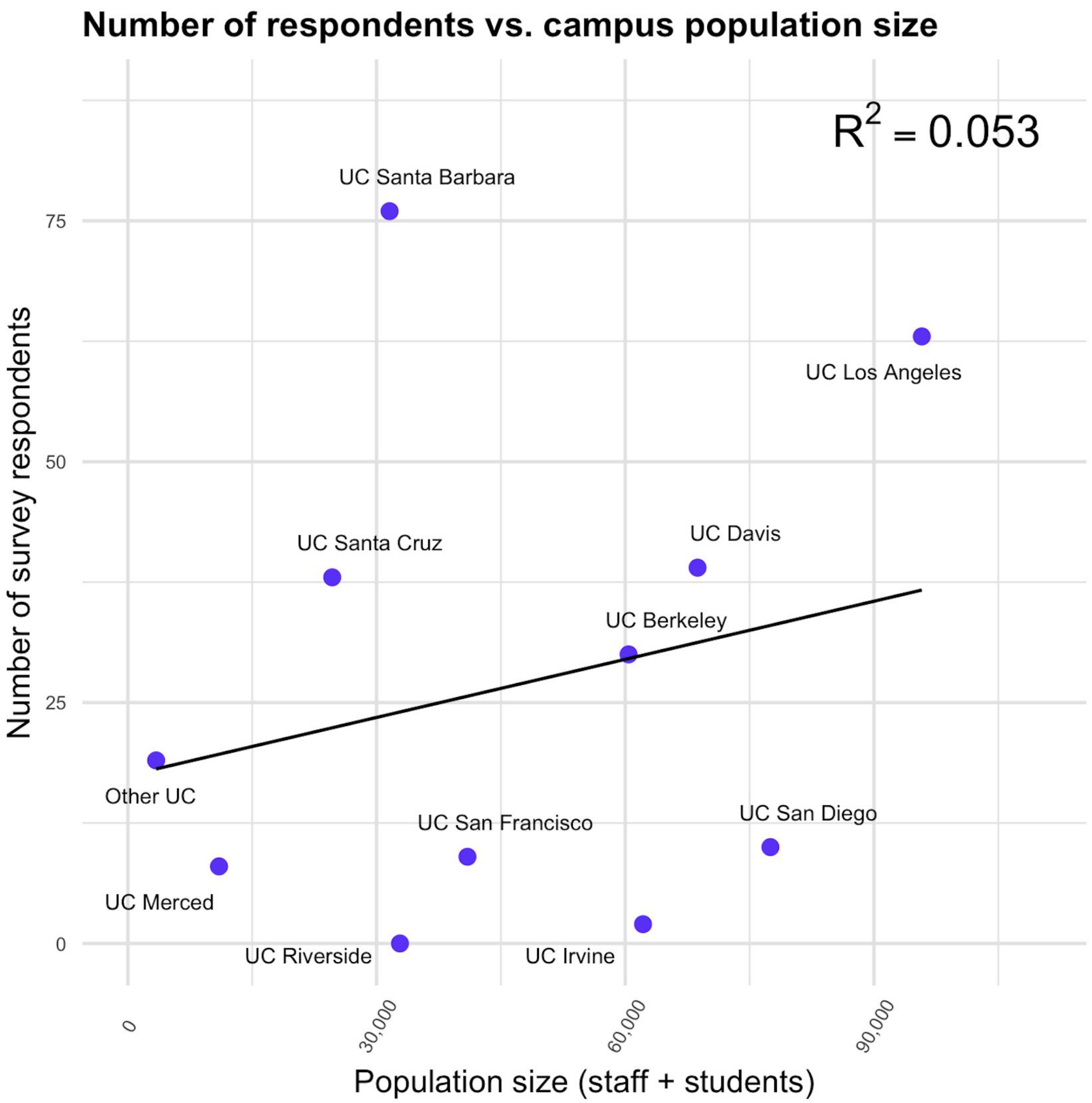

S2 Fig. No correlation between campus size and response rate. Scatterplot showing, for each UC campus, the approximate size of the campus community (staff and students, based on data from the UC Information Center), versus the number of responses received from that campus in this survey. The coefficient of determination for this relationship is shown in the top-right.
