# Supplementary material for: A system-wide snapshot: A multi-campus survey of open source contributors at the University of California
Source: PLoS One. 2026 Jun 5;21(6):e0348894. doi: 10.1371/journal.pone.0348894 (PMC13241014; doi:10.1371/journal.pone.0348894)
Supplement: S3 Fig — UpSet plot showing the number of survey respondents who identified as a Maintainer and/or Contributor and/or Bug reporter (n = 233 total respondents). The third column from the right shows respondents who identified with none of these three roles. Right-side bars show the total number of respondents who selected each role. Note that throughout this report, “contributor” is used as a broad umbrella term, but the term “contributor” for the purposes of this question is more specific, and is defined in the survey instrument Q4 as “Contributing relatively small amounts of code or hardware design, for example by fixing bugs or adding new features.”. (PDF) [file pone.0348894.s004.pdf]

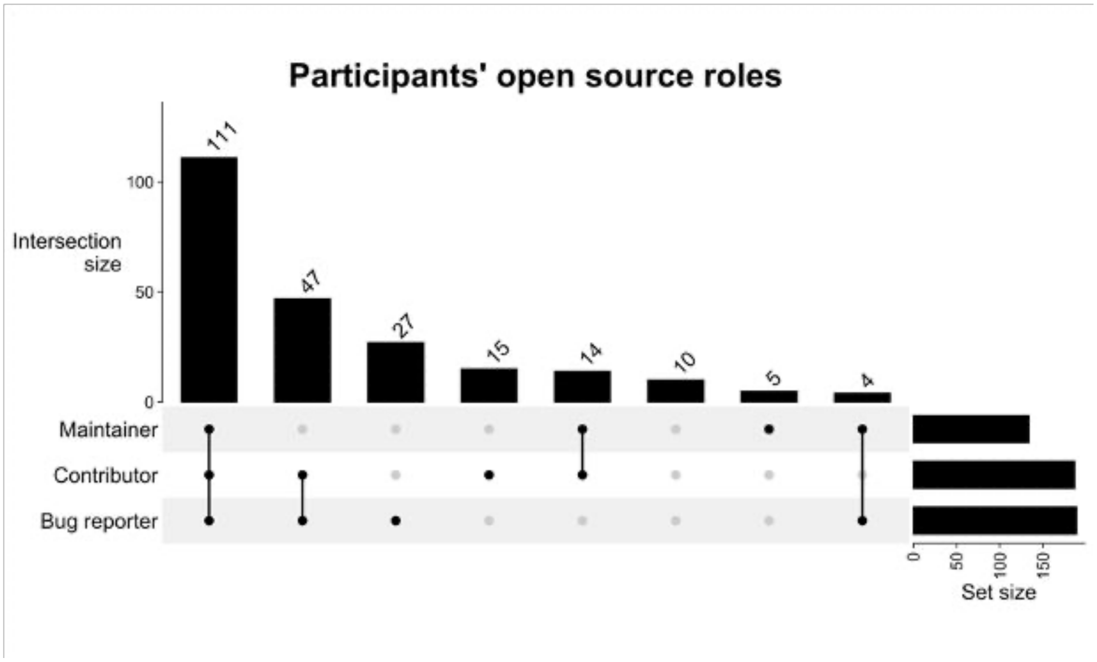

S3 Fig. Maintainer/contributor/bug reporter overlap. UpSet plot showing the number of survey respondents who identified as a Maintainer and/or Contributor and/or Bug reporter (n=233 total respondents). The third column from the right shows respondents who identified with none of these three roles. Right-side bars show the total number of respondents who selected each role. Note that throughout this report, "contributor" is used as a broad umbrella term, but the term "contributor" for the purposes of this question is more specific, and is defined in the survey instrument Q4 as "Contributing relatively small amounts of code or hardware design, for example by fixing bugs or adding new features."
