# Supplementary material for: A system-wide snapshot: A multi-campus survey of open source contributors at the University of California
Source: PLoS One. 2026 Jun 5;21(6):e0348894. doi: 10.1371/journal.pone.0348894 (PMC13241014; doi:10.1371/journal.pone.0348894)
Supplement: S4 Fig — (A) Minimal model accounting only for variation between individuals. Reference level was arbitrarily selected to be ‘Small’. (B) Model that also includes job category as a fixed effect and an interaction between job category and project size. Reference levels were arbitrarily selected to be ‘Students’ and ‘Small’. (PDF) [file pone.0348894.s005.pdf]

A

```
+ summary(fit1.1)
Cumulative Link Mixed Model fitted with the Laplace approximation

formula: frequency ~ size + (1 | participantID)
data:    combined4

 link  threshold nobs logLik  AIC     niter    max.grad cond.H
 logit flexible  699  -901.92 1815.84 324(974) 1.78e-04 3.3e+01

Random effects:
 Groups        Name        Variance Std.Dev.
 participantID (Intercept) 0.6659   0.816
Number of groups:  participantID 233

Coefficients:
           Estimate Std. Error z value Pr(>|z|)
sizeMedium  -1.1717     0.1802  -6.503 7.89e-11 ***
sizeLarge   -2.0412     0.1989 -10.261  < 2e-16 ***
---
Signif. codes:  0 ‘***’ 0.001 ‘**’ 0.01 ‘*’ 0.05 ‘.’ 0.1 ‘ ’ 1

Threshold coefficients:
                                     Estimate Std. Error z value
Never|Relatively infrequently         -2.8019     0.1951 -14.359
Relatively infrequently|Occasionally  -1.2340     0.1548  -7.972
Occasionally|Relatively frequently     0.1129     0.1425   0.792
```

B

```
> summary(fit6)
Cumulative Link Mixed Model fitted with the Laplace approximation

formula: frequency ~ job_category * size + (1 | participantID)
data:    combined4

 link  threshold nobs logLik  AIC     niter      max.grad cond.H
 logit flexible  699  -882.36 1794.73 1371(4115) 7.60e-04 5.9e+02

Random effects:
 Groups        Name        Variance Std.Dev.
 participantID (Intercept) 0.7988   0.8938
Number of groups:  participantID 233

Coefficients:
                                                   Estimate Std. Error z value Pr(>|z|)
job_categoryPostdocs and Staff Researchers           0.1496     0.4840   0.309 0.757160
job_categoryFaculty                                 -0.3564     0.4772  -0.747 0.455136
job_categoryNon-research Staff                      -0.8317     0.4472  -1.860 0.062952 .
sizeMedium                                          -2.4019     0.4875  -4.927 8.37e-07 ***
sizeLarge                                           -3.1399     0.5066  -6.197 5.74e-10 ***
job_categoryPostdocs and Staff Researchers:sizeMedium  1.0664     0.6045   1.764 0.077741 .
job_categoryFaculty:sizeMedium                       0.8673     0.5932   1.462 0.143741
job_categoryNon-research Staff:sizeMedium            1.9299     0.5613   3.438 0.000586 ***
job_categoryPostdocs and Staff Researchers:sizeLarge   0.1753     0.6210   0.282 0.777782
job_categoryFaculty:sizeLarge                        0.5136     0.6102   0.842 0.399954
job_categoryNon-research Staff:sizeLarge             2.2159     0.5748   3.855 0.000116 ***
---
Signif. codes:  0 ‘***’ 0.001 ‘**’ 0.01 ‘*’ 0.05 ‘.’ 0.1 ‘ ’ 1

Threshold coefficients:
                                     Estimate Std. Error z value
Never|Relatively infrequently         -3.2971     0.4143  -7.957
Relatively infrequently|Occasionally  -1.6379     0.3921  -4.177
Occasionally|Relatively frequently    -0.2328     0.3845  -0.605
```

S4 Fig. Ordinal regression of participants’ contribution frequency to projects of a certain size. (A) Minimal model accounting only for variation between individuals. Reference level was arbitrarily selected to be ‘Small’. (B) Model that also includes job category as a fixed effect and an interaction between job category and project size. Reference levels were arbitrarily selected to be ‘Students’ and ‘Small’.
