# Supplementary material for: A system-wide snapshot: A multi-campus survey of open source contributors at the University of California
Source: PLoS One. 2026 Jun 5;21(6):e0348894. doi: 10.1371/journal.pone.0348894 (PMC13241014; doi:10.1371/journal.pone.0348894)
Supplement: S5 Fig — Y-axis shows the percent of eligible respondents in each job category who selected the x-axis value shown. The question was, “How frequently have you contributed to projects of the following size? For each project size, please answer relative to the other sizes.”. (A) Large projects. (B) Small projects. (PDF) [file pone.0348894.s006.pdf]

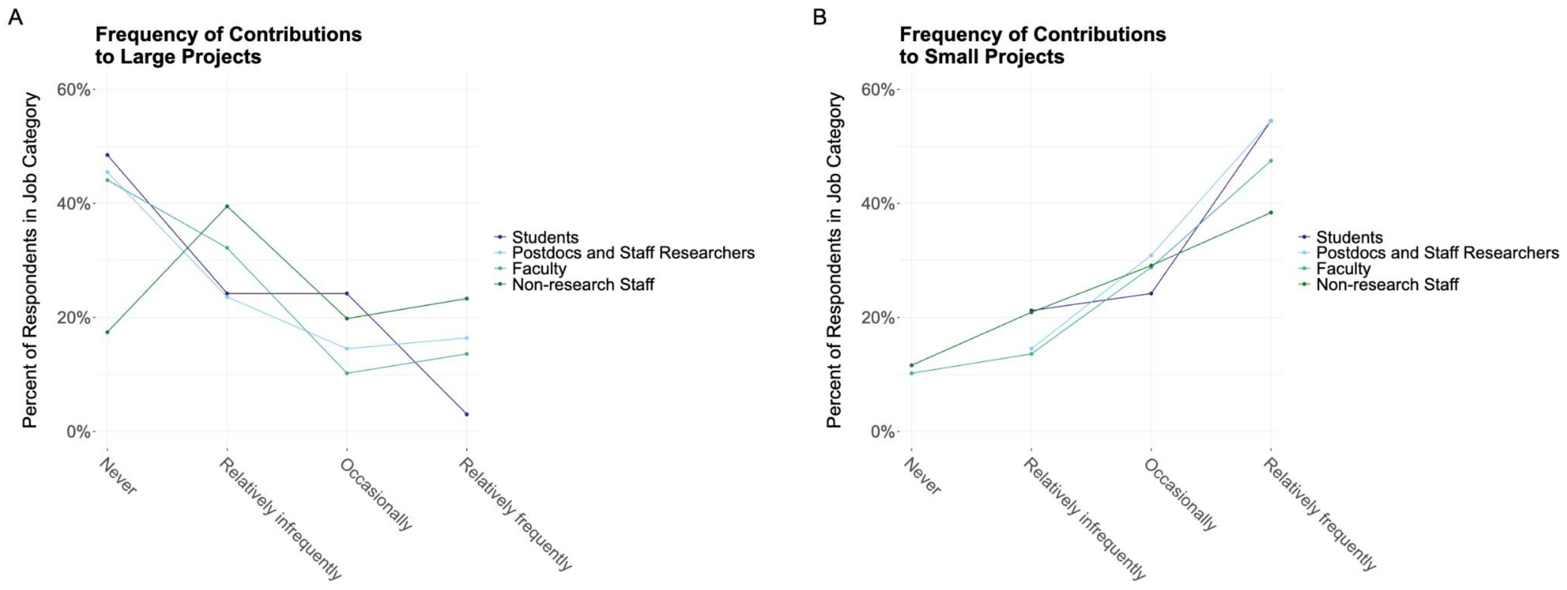

S5 Fig. Frequency of contributions to large and small projects. Y-axis shows the percent of eligible respondents in each job category who selected the x-axis value shown. The question was, “How frequently have you contributed to projects of the following size? For each project size, please answer relative to the other sizes.”. (A) Large projects. (B) Small projects.
