# Supplementary material for: A system-wide snapshot: A multi-campus survey of open source contributors at the University of California
Source: PLoS One. 2026 Jun 5;21(6):e0348894. doi: 10.1371/journal.pone.0348894 (PMC13241014; doi:10.1371/journal.pone.0348894)
Supplement: S6 Fig — Horizontal axis indicates the percent of respondents in each job category who reported having contributed to the project type on the vertical axis. (PDF) [file pone.0348894.s007.pdf]

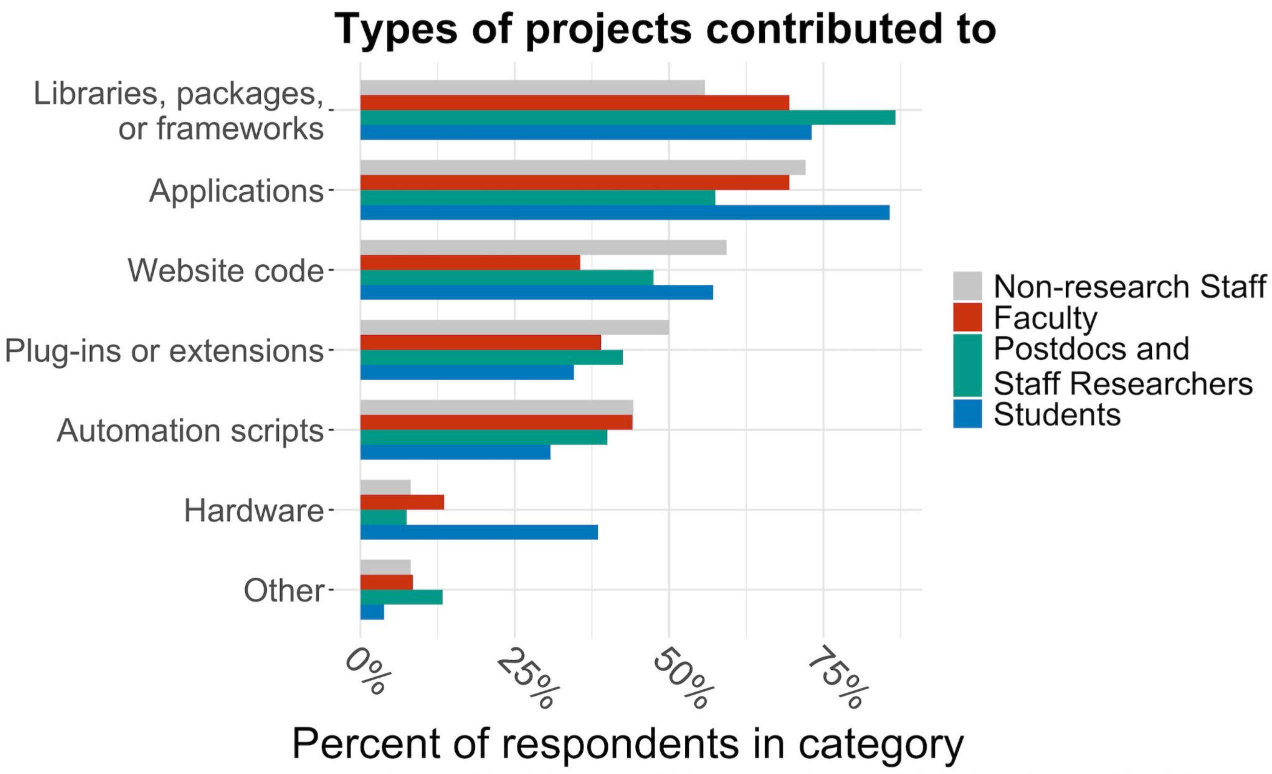

S6 Fig. Contributions to different project types by job category. Horizontal axis indicates the percent of respondents in each job category who reported having contributed to the project type on the vertical axis.
