# Supplementary material for: A system-wide snapshot: A multi-campus survey of open source contributors at the University of California
Source: PLoS One. 2026 Jun 5;21(6):e0348894. doi: 10.1371/journal.pone.0348894 (PMC13241014; doi:10.1371/journal.pone.0348894)
Supplement: S7 Fig — (A) Percent of respondents in each field of study who reported having ever used the platform on the x-axis. (Limited to academics.) (B) Percent of respondents from each UC campus who reported having ever used the platform on the x-axis. (C) Percent of respondents from each UC campus who reported ever having used the data repository on the x-axis. Note that numbers do not necessarily add up to 100% because participants could have selected all or none of these options. (PDF) [file pone.0348894.s008.pdf]

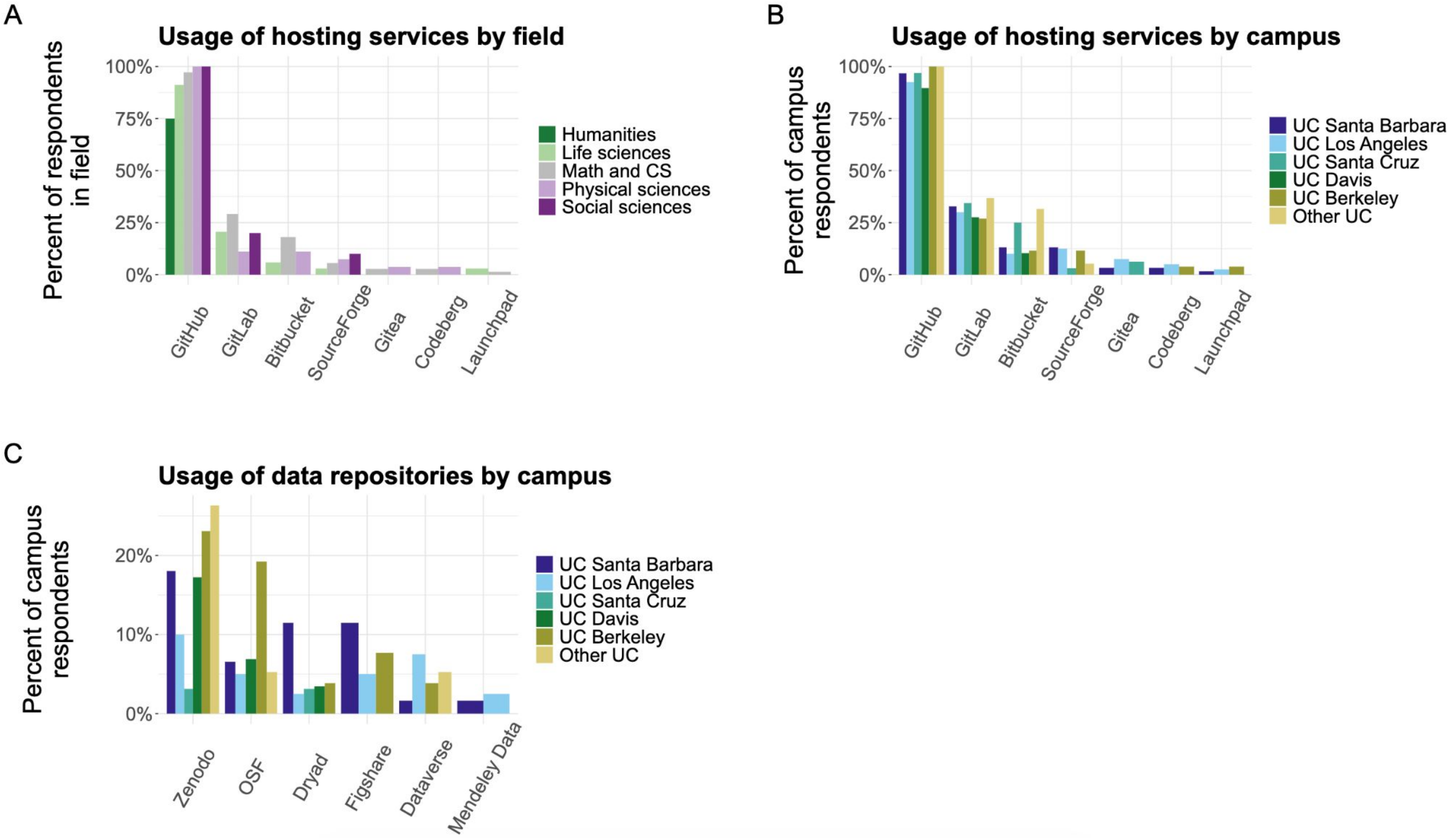

S7 Fig. Usage of code/data sharing platforms. (A) Percent of respondents in each field of study who reported having ever used the platform on the x-axis. (Limited to academics.) (B) Percent of respondents from each UC campus who reported having ever used the platform on the x-axis. (C) Percent of respondents from each UC campus who reported ever having used the data repository on the x-axis. Note that numbers do not necessarily add up to 100% because participants could have selected all or none of these options.
