# Supplementary material for: A system-wide snapshot: A multi-campus survey of open source contributors at the University of California
Source: PLoS One. 2026 Jun 5;21(6):e0348894. doi: 10.1371/journal.pone.0348894 (PMC13241014; doi:10.1371/journal.pone.0348894)
Supplement: S8 Fig — (A) Mean rating after coding rating scale responses to numeric values (e.g., “Never” = 0, “Rarely” = 1, etc.). (B) Percent of respondents in each job category who selected “Frequently” or “Always”. (PDF) [file pone.0348894.s009.pdf]

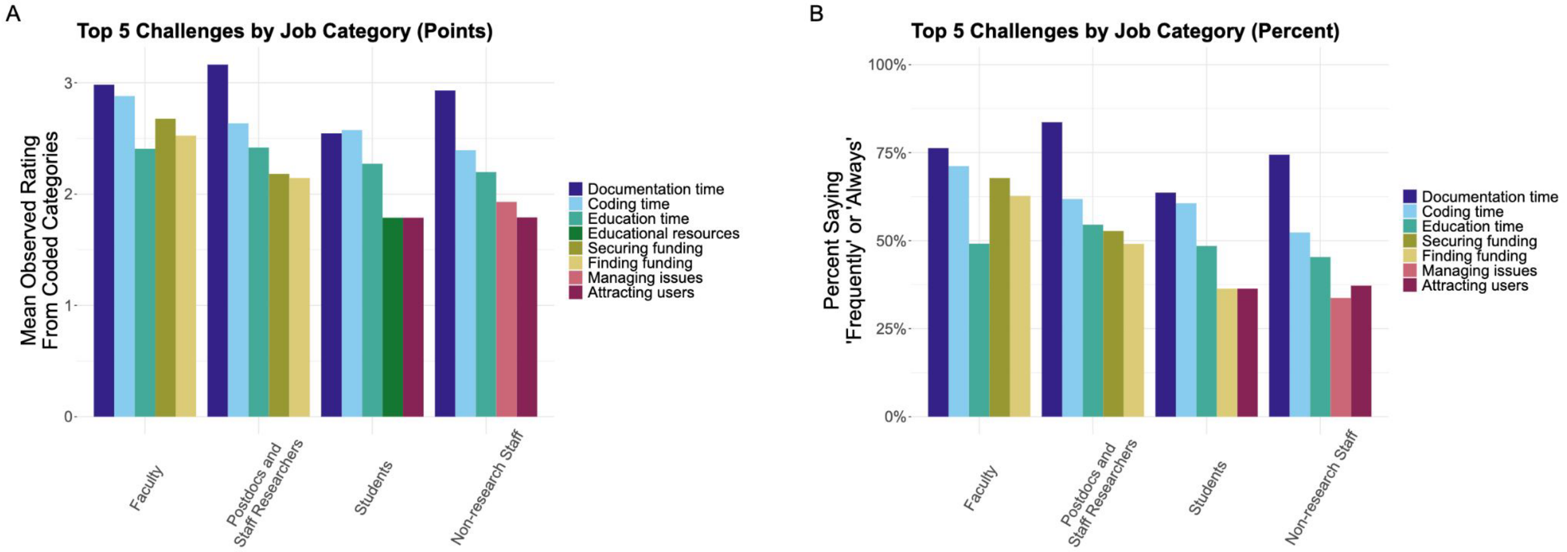

S8 Fig. Top five challenges for each job category, according to two different methods, from data in our survey population. (A) Mean rating after coding rating scale responses to numeric values (e.g. "Never" = 0, "Rarely" = 1, etc.). (B) Percent of respondents in each job category who selected “Frequently” or “Always”.
