# Supplementary material for: A system-wide snapshot: A multi-campus survey of open source contributors at the University of California
Source: PLoS One. 2026 Jun 5;21(6):e0348894. doi: 10.1371/journal.pone.0348894 (PMC13241014; doi:10.1371/journal.pone.0348894)
Supplement: S9 Fig — (The cluster 2 challenges are ‘Finding funding’, ‘Securing funding’, and ‘Hiring’.) (A) Output from ordinal regression of challenge frequency ratings on job categories and cluster 2 challenges. Reference levels were arbitrarily selected to be “Faculty” and ‘Finding funding’. (B) Estimated marginal means from regression in a). (C) Significantly different job category comparisons according to a Dunn’s test, for each cluster 2 challenge. (PDF) [file pone.0348894.s010.pdf]

A

```
> summary(fit5)
Cumulative Link Mixed Model fitted with the Laplace approximation

formula: challenge_level ~ job_category + challenge + (1 | participantID)
data:    cluster2data

 link  threshold nobs logLik   AIC     niter     max.grad cond.H
 logit flexible  699  -1032.39 2086.78 899(4370) 2.31e-05 3.7e+02

Random effects:
 Groups        Name        Variance Std.Dev.
 participantID (Intercept) 2.59     1.609
Number of groups:  participantID 233

Coefficients:
                                          Estimate Std. Error z value Pr(>|z|)
job_categoryNon-research Staff              1.4796     0.3426   4.319 1.56e-05 ***
job_categoryPostdocs and Staff Researchers   0.7994     0.3761   2.126 0.033541 *
job_categoryStudents                        1.4941     0.4329   3.451 0.000558 ***
challengeHiring                             0.6703     0.1812   3.698 0.000217 ***
challengeSecuring funding                  -0.1825     0.1763  -1.035 0.300724
---
Signif. codes:  0 ‘***’ 0.001 ‘**’ 0.01 ‘*’ 0.05 ‘.’ 0.1 ‘ ’ 1

Threshold coefficients:
                            Estimate Std. Error z value
Always|Frequently            -0.7551     0.2887  -2.615
Frequently|Never              0.5245     0.2891   1.815
Never|Non-applicable          0.9640     0.2926   3.294
Non-applicable|Occasionally   3.5721     0.3308  10.799
Occasionally|Rarely           4.8889     0.3676  13.299
>
```

B

```
> emm2 <- emmeans(fit5, ~ job_category | challenge, mode = "mean.class")
+ by_chall <- summary(
+   pairs(emm2, by = "challenge"),
+   infer = TRUE # infer CIs
+ )
+
+ by_chall
challenge = Finding funding:
 contrast                                          estimate    SE  df asymp.LCL asymp.UCL z.ratio p.value
 Faculty - (Non-research Staff)                    -1.03047 0.227 Inf    -1.613    -0.448  -4.542  <.0001
 Faculty - Postdocs and Staff Researchers          -0.56513 0.261 Inf    -1.237     0.107  -2.162  0.1341
 Faculty - Students                                -1.03984 0.284 Inf    -1.770    -0.309  -3.657  0.0015
 (Non-research Staff) - Postdocs and Staff Researchers  0.46535 0.233 Inf    -0.134     1.065   1.994  0.1900
 (Non-research Staff) - Students                   -0.00936 0.258 Inf    -0.673     0.654  -0.036  1.0000
 Postdocs and Staff Researchers - Students         -0.47471 0.289 Inf    -1.218     0.269  -1.640  0.3560

challenge = Hiring:
 contrast                                          estimate    SE  df asymp.LCL asymp.UCL z.ratio p.value
 Faculty - (Non-research Staff)                    -0.96846 0.225 Inf    -1.546    -0.391  -4.310  0.0001
 Faculty - Postdocs and Staff Researchers          -0.55074 0.256 Inf    -1.208     0.107  -2.151  0.1371
 Faculty - Students                                -0.97680 0.272 Inf    -1.675    -0.279  -3.596  0.0018
 (Non-research Staff) - Postdocs and Staff Researchers  0.41772 0.212 Inf    -0.128     0.963   1.968  0.2002
 (Non-research Staff) - Students                   -0.00834 0.230 Inf    -0.599     0.582  -0.036  1.0000
 Postdocs and Staff Researchers - Students         -0.42606 0.261 Inf    -1.098     0.246  -1.630  0.3617

challenge = Securing funding:
 contrast                                          estimate    SE  df asymp.LCL asymp.UCL z.ratio p.value
 Faculty - (Non-research Staff)                    -1.03296 0.225 Inf    -1.611    -0.455  -4.589  <.0001
 Faculty - Postdocs and Staff Researchers          -0.55827 0.259 Inf    -1.223     0.106  -2.159  0.1349
 Faculty - Students                                -1.04261 0.286 Inf    -1.778    -0.307  -3.643  0.0015
 (Non-research Staff) - Postdocs and Staff Researchers  0.47469 0.237 Inf    -0.134     1.083   2.005  0.1862
 (Non-research Staff) - Students                   -0.00965 0.266 Inf    -0.693     0.674  -0.036  1.0000
 Postdocs and Staff Researchers - Students         -0.48434 0.295 Inf    -1.243     0.275  -1.640  0.3563

Confidence level used: 0.95
Conf-level adjustment: tukey method for comparing a family of 4 estimates
P value adjustment: tukey method for comparing a family of 4 estimates
```

C

```
> pairwise_results <- lapply(unique(cluster2data_numcoded$challenge), function(chall) {
+   df <- subset(cluster2data_numcoded, challenge == chall)
+   out <- FSA::dunnTest(challenge_score ~ job_category, data = df, method = "bh")
+   cbind(challenge = chall, out$res)
+ })
+ pairwise_results <- do.call(rbind, pairwise_results)
> subset(pairwise_results, P.adj < 0.05)
          challenge                                     Comparison         Z       P.unadj         P.adj
1            Hiring                   Faculty - Non-research Staff  3.202369 1.363021e-03 4.089062e-03
2            Hiring       Faculty - Postdocs and Staff Researchers  2.483752 1.300062e-02 2.600123e-02
4            Hiring                             Faculty - Students  4.191500 2.771168e-05 1.662701e-04
7   Finding funding                   Faculty - Non-research Staff  4.319614 1.563026e-05 9.378155e-05
9   Finding funding Non-research Staff - Postdocs and Staff Researchers -2.899242 3.740657e-03 1.122197e-02
10  Finding funding                             Faculty - Students  2.664241 7.716231e-03 1.543246e-02
13 Securing funding                   Faculty - Non-research Staff  4.741135 2.125239e-06 1.275143e-05
15 Securing funding Non-research Staff - Postdocs and Staff Researchers -3.077589 2.086828e-03 4.173655e-03
16 Securing funding                             Faculty - Students  3.174243 1.502280e-03 4.506841e-03
```

S9 Fig. Statistics demonstrating faculty’s preferential rating of cluster 2 challenges. (The cluster 2 challenges are ‘Finding funding’, ‘Securing funding’, and ‘Hiring’.) (A) Output from ordinal regression of challenge frequency ratings on job categories and cluster 2 challenges. Reference levels were arbitrarily selected to be “Faculty” and ‘Finding funding’. (B) Estimated marginal means from regression in a). (C) Significantly different job category comparisons according to a Dunn’s test, for each cluster 2 challenge.
