# Supplementary material for: A system-wide snapshot: A multi-campus survey of open source contributors at the University of California
Source: PLoS One. 2026 Jun 5;21(6):e0348894. doi: 10.1371/journal.pone.0348894 (PMC13241014; doi:10.1371/journal.pone.0348894)
Supplement: S10 Fig — (A) Mean rating after coding rating scale responses to numeric values (“Not very useful” = 0, “Useful” = 1, “Very useful” = 2). (B) Percent of respondents in each job category who selected “Useful” or “Very useful”. (C) Top five solutions from Q11, where participants had to choose their favorite solution, in terms of the number of participants who chose that solution. (PDF) [file pone.0348894.s011.pdf]

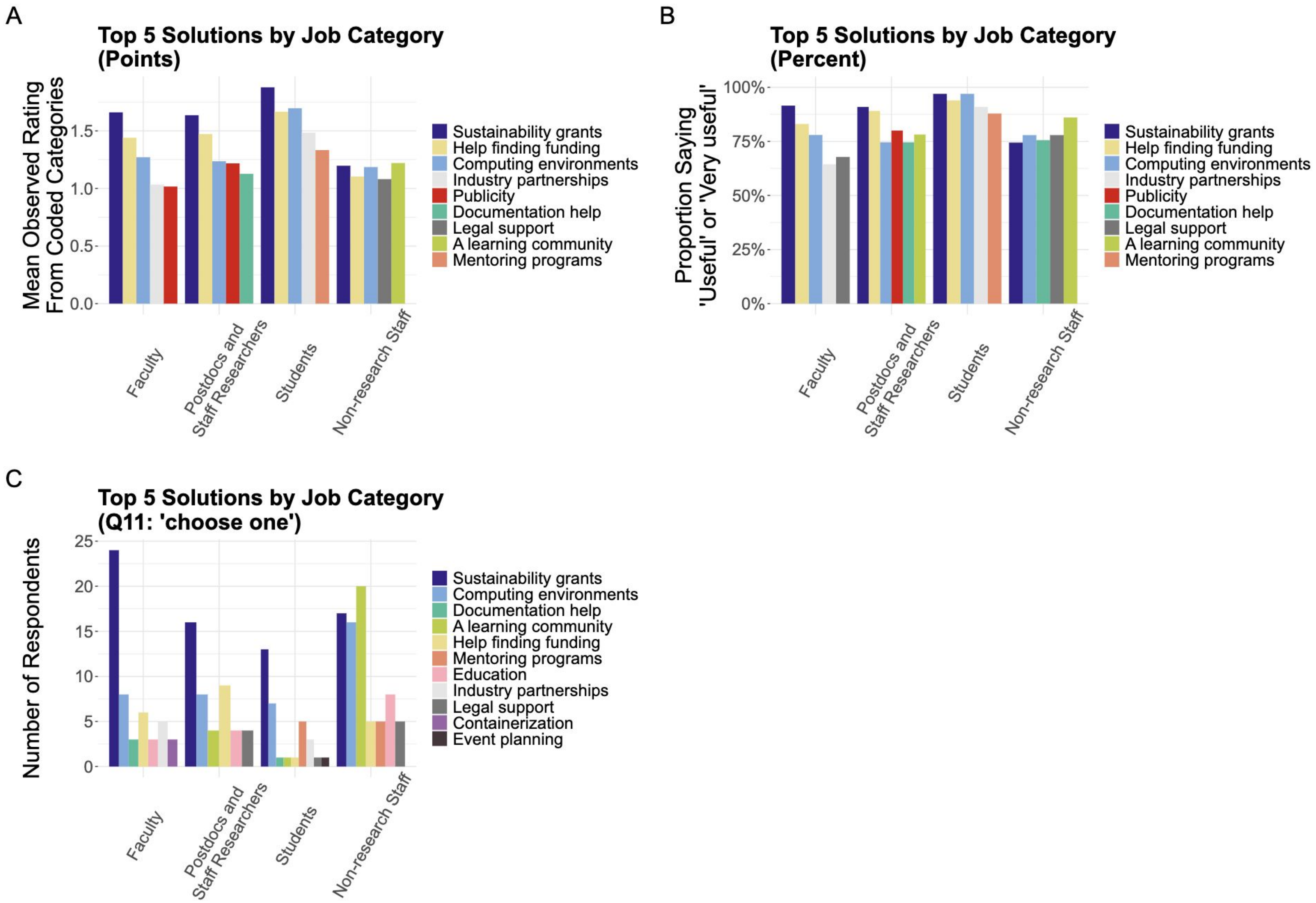

S10 Fig. Top five solutions for each job category, as observed in our survey population, according to several different methods. (A) Mean rating after coding rating scale responses to numeric values ("Not very useful" = 0, "Useful" = 1, "Very useful" = 2). (B) Percent of respondents in each job category who selected "Useful" or "Very useful". (C) Top five solutions from Q11, where participants had to choose their favorite solution, in terms of the number of participants who chose that solution.
