# Supplementary material for: A system-wide snapshot: A multi-campus survey of open source contributors at the University of California
Source: PLoS One. 2026 Jun 5;21(6):e0348894. doi: 10.1371/journal.pone.0348894 (PMC13241014; doi:10.1371/journal.pone.0348894)
Supplement: S11 Fig — Participants rated the utility of each solution on a scale of “Not very useful”, “Useful”, “Very useful”. (A) Output from minimal ordinal regression model of utility ratings on solutions, with participant as a random effect. Reference level was arbitrarily selected to be ‘Computing environments’. (B) Output from ordinal regression of utility ratings on job categories and solutions, with an interaction between the two. Reference levels were arbitrarily selected to be “Faculty” and ‘Computing environments’. (PDF) [file pone.0348894.s012.pdf]

A

```
+ summary(fit0)
Cumulative Link Mixed Model fitted with the Laplace approximation

formula: utility ~ solution + (1 | participantID)
data:    long_data

 link  threshold nobs logLik   AIC     niter      max.grad cond.H
 logit flexible  2602 -2404.03 4836.07 1430(5759) 3.88e-03 1.5e+02

Random effects:
 Groups        Name        Variance Std.Dev.
 participantID (Intercept) 2.154    1.468
Number of groups:  participantID 232

Coefficients:
                              Estimate Std. Error z value Pr(>|z|)
solutionPublicity              -0.7980     0.2022  -3.946 7.94e-05 ***
solutionContainerization       -1.2881     0.2025  -6.362 2.00e-10 ***
solutionDocumentation help     -0.8078     0.1978  -4.084 4.43e-05 ***
solutionA learning community   -0.7697     0.1946  -3.955 7.67e-05 ***
solutionEvent planning         -1.4711     0.2032  -7.239 4.51e-13 ***
solutionMentoring programs     -1.1196     0.1986  -5.637 1.73e-08 ***
solutionEducation              -0.9347     0.1985  -4.710 2.48e-06 ***
solutionLegal support          -0.7838     0.1984  -3.951 7.79e-05 ***
solutionIndustry partnerships  -0.7661     0.2038  -3.759 0.000171 ***
solutionSustainability grants   1.2038     0.2201   5.468 4.55e-08 ***
solutionHelp finding funding    0.5753     0.2098   2.742 0.006111 **
---
Signif. codes:  0 ‘***’ 0.001 ‘**’ 0.01 ‘*’ 0.05 ‘.’ 0.1 ‘ ’ 1

Threshold coefficients:
                       Estimate Std. Error z value
Not very useful|Useful -2.28297    0.18164 -12.569
Useful|Very useful     -0.02137    0.17425  -0.123
```

B

```
> summary(fit1b)
Cumulative Link Mixed Model fitted with the Laplace approximation

formula: utility ~ job_category * solution + (1 | participantID)
data:    combined

 link  threshold nobs logLik   AIC     niter        max.grad cond.H
 logit flexible  2602 -2350.98 4801.97 10197(40741) 1.22e-03 2.8e+03

Random effects:
 Groups        Name        Variance Std.Dev.
 participantID (Intercept) 2.097    1.448
Number of groups:  participantID 232

Coefficients:
                                                                  Estimate Std. Error z value Pr(>|z|)
job_categoryPostdocs and Staff Researchers                        -0.04736    0.49392  -0.096 0.923611
job_categoryStudents                                               1.66906    0.61824   2.700 0.006941 **
job_categoryNon-research Staff                                    -0.08350    0.44158  -0.189 0.850023
solutionPublicity                                                 -0.66811    0.40568  -1.647 0.099586 .
solutionContainerization                                          -1.06243    0.40387  -2.631 0.008524 **
solutionDocumentation help                                        -1.21045    0.39881  -3.035 0.002404 **
solutionA learning community                                      -1.56910    0.38854  -4.038 5.38e-05 ***
solutionEvent planning                                            -1.83275    0.40245  -4.554 5.26e-06 ***
solutionMentoring programs                                        -1.93070    0.39813  -4.849 1.24e-06 ***
solutionEducation                                                 -1.68608    0.39749  -4.242 2.22e-05 ***
solutionLegal support                                             -1.18188    0.39142  -3.019 0.002532 **
solutionIndustry partnerships                                     -0.68400    0.40142  -1.704 0.088390 .
solutionSustainability grants                                      1.73451    0.44904   3.863 0.000112 ***
solutionHelp finding funding                                       1.08082    0.42632   2.535 0.011237 *
job_categoryPostdocs and Staff Researchers:solutionPublicity       0.78228    0.57881   1.352 0.176523
job_categoryStudents:solutionPublicity                            -0.49909    0.72018  -0.693 0.488306
job_categoryNon-research Staff:solutionPublicity                  -0.74669    0.52227  -1.430 0.152797
job_categoryPostdocs and Staff Researchers:solutionContainerization -0.03388  0.57095  -0.059 0.952681
job_categoryStudents:solutionContainerization                     -0.64866    0.71859  -0.903 0.366692
job_categoryNon-research Staff:solutionContainerization           -0.42332    0.52130  -0.812 0.416773
job_categoryPostdocs and Staff Researchers:solutionDocumentation help  0.94435 0.57355 1.646 0.099662 .
job_categoryStudents:solutionDocumentation help                   -0.35912    0.70165  -0.512 0.608771
job_categoryNon-research Staff:solutionDocumentation help          0.53196    0.50803   1.047 0.295052
job_categoryPostdocs and Staff Researchers:solutionA learning community  1.02260 0.55805 1.832 0.066882 .
job_categoryStudents:solutionA learning community                 -0.10889    0.69278  -0.157 0.875099
job_categoryNon-research Staff:solutionA learning community        1.44068    0.49959   2.884 0.003930 **
job_categoryPostdocs and Staff Researchers:solutionEvent planning  0.91886    0.57443   1.600 0.109686
job_categoryStudents:solutionEvent planning                       -0.22759    0.71107  -0.320 0.748914
job_categoryNon-research Staff:solutionEvent planning              0.35351    0.51964   0.680 0.496319
job_categoryPostdocs and Staff Researchers:solutionMentoring programs  1.04164 0.57321 1.817 0.069187 .
job_categoryStudents:solutionMentoring programs                    0.45731    0.69274   0.660 0.509161
job_categoryNon-research Staff:solutionMentoring programs          1.24157    0.51079   2.431 0.015071 *
job_categoryPostdocs and Staff Researchers:solutionEducation       1.16078    0.57092   2.033 0.042035 *
job_categoryStudents:solutionEducation                             0.19222    0.69830   0.275 0.783112
job_categoryNon-research Staff:solutionEducation                   1.12431    0.51032   2.203 0.027585 *
job_categoryPostdocs and Staff Researchers:solutionLegal support   0.54219    0.56944   0.952 0.341017
job_categoryStudents:solutionLegal support                        -0.35340    0.69631  -0.508 0.611776
job_categoryNon-research Staff:solutionLegal support               0.77637    0.50550   1.536 0.124579
job_categoryPostdocs and Staff Researchers:solutionIndustry partnerships  0.34019 0.58203 0.584 0.558895
job_categoryStudents:solutionIndustry partnerships                -0.23308    0.71261  -0.327 0.743610
job_categoryNon-research Staff:solutionIndustry partnerships      -0.46161    0.52272  -0.883 0.377183
job_categoryPostdocs and Staff Researchers:solutionSustainability grants -0.05181 0.63964 -0.081 0.935446
job_categoryStudents:solutionSustainability grants                 0.28703    0.99068   0.290 0.772020
job_categoryNon-research Staff:solutionSustainability grants      -1.21278    0.56400  -2.150 0.031531 *
job_categoryPostdocs and Staff Researchers:solutionHelp finding funding -0.02601 0.60587 -0.043 0.965754
job_categoryStudents:solutionHelp finding funding                 -0.76619    0.77727  -0.986 0.324256
job_categoryNon-research Staff:solutionHelp finding funding       -1.03639    0.54190  -1.912 0.055813 .
---
Signif. codes:  0 ‘***’ 0.001 ‘**’ 0.01 ‘*’ 0.05 ‘.’ 0.1 ‘ ’ 1

Threshold coefficients:
                       Estimate Std. Error z value
Not very useful|Useful  -2.1535     0.3461  -6.223
Useful|Very useful       0.1717     0.3425   0.501
>
```

S11 Fig. Regression of participants’ ratings of solutions. Participants rated the utility of each solution on a scale of “Not very useful”, “Useful”, “Very useful”. (A) Output from minimal ordinal regression model of utility ratings on solutions, with participant as a random effect. Reference level was arbitrarily selected to be ‘Computing environments’. (B) Output from ordinal regression of utility ratings on job categories and solutions, with an interaction between the two. Reference levels were arbitrarily selected to be “Faculty” and ‘Computing environments’.
